# Human lactoferrin induces asthmatic symptoms in NC/Nga mice

**DOI:** 10.14814/phy2.13365

**Published:** 2017-08-03

**Authors:** Kenjiro Nagaoka, Tatsuo Ito, Keiki Ogino, Eri Eguchi, Yoshihisa Fujikura

**Affiliations:** ^1^ Department of Public Health Okayama University Graduate School of Medicine, Dentistry and Pharmaceutical Sciences Okayama Japan; ^2^ Department of Molecular Anatomy Faculty of Medicine Oita University Oita Japan

**Keywords:** Asthma, bronchus, human lactoferrin, inflammation, lung

## Abstract

Lactoferrin in commercial supplements is known to exert anti‐viral and anti‐allergic effects. However, this is the first study to evaluate the induction of allergic airway inflammation in NC/Nga mice. Human lactoferrin was administered intraperitoneally with aluminum oxide for sensitization. Five days later, lactoferrin was inoculated intranasally for 5 days, and then on the 12th day, the single inoculation of lactoferrin intranasally was performed as a challenge. On the 13th day, airway hypersensitivity was assessed (AHR), a bronchoalveolar fluid (BALF) cell analysis was conducted, serum IgE and serum lactoferrin‐specific IgG and IgE levels as well as the mRNA expression levels of cytokines and chemokines in the lung were measured, and a histopathological analysis of the lung was performed. Human lactoferrin increased AHR, the number of eosinophils in BALF, serum lactoferrin‐specific IgG levels, and the mRNA levels of IL‐13, eotaxin 1, and eotaxin 2. Moreover, the accumulation of inflammatory cells around the bronchus and the immunohistochemical localization of arginase I and human lactoferrin were detected. Collectively, these results indicate that human lactoferrin induced allergic airway inflammation in mice. Therefore, the commercial use of human lactoferrin in supplements warrants more intensive study.

## Introduction

Lactoferrin in supplements is known to exert anti‐bacterial and anti‐viral effects. Lactoferrin is one of the main proteins found in the breast milk of humans and cows and has been detected in various tissues including neutrophil, secondary granules, lacrimal fluid, colostrum, saliva, and mucosal secretions (Ward et al. 2005). Lactoferrin is an 80‐kDa iron‐binding glycoprotein (Lönnerdal and Iyer 1995), belongs to the transferrin family, and has various functions such as the scavenging/transport of iron, stimulation of neutrophil aggregation (Oseas et al. 1981), inhibition of eosinophil migration (Bournazou et al. 2010), generation of superoxide ($O_{2}^{-}$) by eosinophils (Thomas et al. 2002), enhancement of NK cell activity (Damiens et al. 1998), inhibition of intestinal immune responses (Li et al. 2014), and anti‐cancer effects (Deng et al. 2013).

Previous studies reported that lactoferrin ameliorated the severity of allergies and inflammation (Wang et al. 2013; Hill and Newburg 2015; Giansanti et al. 2016). The amelioration of inflammation has been attributed to decreases in the generation of O_2_^‐^ (Britigan et al. 1994) and the down‐regulated expression of TNF*α* (Baveye et al. 1999), IL‐1*β* (Legrand et al. 2004), and IL‐6 and IL‐8 (Zimecki et al. 1998). However, native human lactoferrin had antigenicity in mice and generates specific IgG, in which iron binding capacity or its glycosylation may contribute to its antigenicity (Almond et al. 2012, 2013). When lactoferrin from human or other animal origins is administered into blood or mucosal tissue, it may become an allergen. Previous studies demonstrated the anti‐asthmatic or anti‐inflammatory effects of lactoferrin, and this study is the first to demonstrate the induction of allergic airway inflammation through the nasal inoculation of NC/Nga mice, which are hypersensitive to mite allergens, with human lactoferrin. We also described the mechanisms underlying allergic airway inflammation in mice in detail.

## Materials and Methods

### Animals

Male NC/Nga mice (7 weeks old, seven mice/group) were purchased from Charles River Laboratories Japan (Yokohama, Japan). Mice were maintained under specific pathogen‐free conditions with a 12‐h light/dark cycle and had free access to a standard diet and tap water. They were acclimatized for at least 1 week before experiments. The care and handling of mice were in accordance with the Guidelines for the Care and Use of Laboratory Animals at the Shikata Campus of Okayama University and approved by the Okayama University Institutional Animal Care and Use Committee.

### Induction of asthma

**Figure 1:**
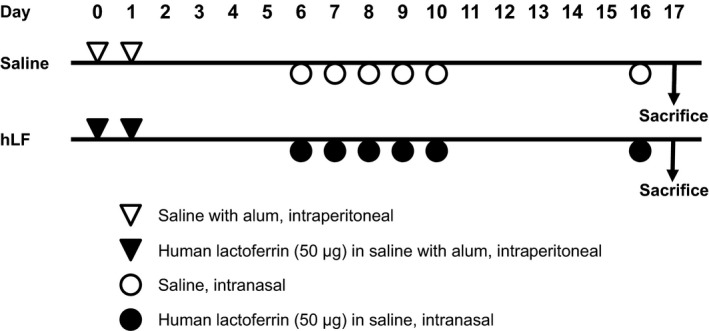
Schematic representation of the experiment. Each mouse group received the mixture with alum intraperitoneally on days 0 and 1. On days 6–10 and 16, intranasal administration was performed. 50 *μ*g of human lactoferrin was dissolved in saline.

Mice were sensitized using intraperitoneal injections of 50 *μ*g human lactoferrin (Sigma, St. Louis, MO) mixed 1:1 with 100 *μ*L Imject Alum (Thermo Scientific, Rockford, IL) on days 0 and 1. This was followed by the intranasal administration of 50 *μ*g human lactoferrin in 25 *μ*L saline under anesthesia with 40 mg/kg sodium pentobarbital (Kyoritsu Seiyaku, Tokyo, Japan) on days 6–10. On day 16, 50 *μ*g human lactoferrin was intranasally administered as a challenge. Instead of human lactoferrin, saline was used as the control group. A schematic protocol is shown in Figure 1.

### Analysis of airway hyperresponsiveness

Analysis of airway hyperresponsiveness (AHR) to acetylcholine (ACh) was measured on day 17, as described previously (Shibamori et al. 2006). Briefly, mice were anesthetized with pentobarbital and connected to an artificial ventilator through a surgical incision in the trachea. A pulmotor system, conducted with a rodent ventilator (Respirator model SN‐480‐7; Shimano Manufacturing Co., Ltd., Tokyo, Japan), was connected to ventilated mice (0.7 mL/stroke, 60 strokes/min). Spontaneous respiration was inhibited by an intravenous injection of gallamine triethiodide (350 *μ*g/mouse, Sigma), followed by the intravenous administration of ACh with stepwise increases in concentration from 62.5 to 4000 *μ*g/kg. Bronchoconstriction was expressed as a percentage of the maximum overflow volume by completely occluding the tracheal cannula.

### Analysis of cells in bronchoalveolar lavage fluid

Briefly, bronchoalveolar lavage fluid (BALF) was collected with 1.0 mL of saline using a trachea cannula after the analysis of AHR. BALF was centrifuged, and cell pellets were resuspended in 1 mL of PBS. Total BALF cell counts were obtained with a hemocytometer and placed on slides with Cytospin. Slides were fixed under natural air, and cells were stained with Wright‐Giemsa. A blind observer assessed the percentage of BALF cells (macrophages, eosinophils, neutrophils, and lymphocytes) on each slide by counting a minimum of 200 cells in random high‐power fields with a light microscope (Takemoto et al. 2007).

### Quantitative real‐time PCR (qRT‐PCR) analysis

Total RNA was isolated from the lungs using ISOGEN (Nippon Gene, Tokyo, Japan). Oligo(dT)‐primed reverse transcription was performed using 1 *μ*g total RNA with a 1st strand cDNA synthesis kit (TaKaRa Bio, Kyoto, Japan) according to the supplier's protocol. A real‐time PCR analysis was performed using StepOnePlus (Applied Biosystems, Foster City, CA). The primer sequences used are shown in Table 1. GAPDH (Glyceraldehyde 3‐phosphate dehydrogenase) was used as an internal control (Ogino et al. 2014, 2016).

**Table 1:** Primers used for Quantitative real‐time PCR

Target gene	Sense primer (5′ to 3′)	Antisense primer (5′ to 3′)
GAPDH	AGGTCGGTGTGAACGGATTTG	TGTAGACCATGTAGTTGAGGTCA
IFN‐*γ*	ATGAACGCTACACACTGCATC	CCATCCTTTTGCCAGTTCCTC
IL‐1*β*	GCAACTGTTCCTGAACTCAACT	ATCTTTTGGGGTCCGTCAACT
IL‐4	TGAACGAGGTCACAGGAGAA	CGAGCTCACTCTCTGTGGTG
IL‐5	CTCTGTTGACAAGCAATGAGACG	TCTTCAGTATGTCTAGCCCCTG
IL‐13	TGTGTCTCTCCCTCTGACCC	CACACTCCATACCATGCTGC
IL‐33	ATCACGGCAGAATCATCGAG	GCGGTGCTGCTGAACTTT
CCL11 (eotaxin 1)	GAATCACCAACAACAGATGCAC	ATCCTGGACCCACTTCTTCTT
CCL24 (eotaxin 2)	AATTCCAGAAAACCGAGTGG	TCTTATGGCCCTTCTTGGTG
ARG1	CTCCAAGCCAAAGTCCTTAGAG	AGGAGCTGTCATTAGGGACATC
ARG2	TCCTCCACGGGCAAATTCC	GCTGGACCATATTCCACTCCTA
NOS1	CTTCAATGATCTGTGGGGGA	GGACTGCCATTCTTGGTAGG
NOS2	GTTCTCAGCCCAACAATACAAGA	GTGGACGGGTCGATGTCAC
NOS3	GACCAGCACATTTGGCAAT	CCTAGGGGAGCTGTTGTACG
MUC5AC	CAGGACTCTCTGAAATCGTACCA	GAAGGCTCGTACCACAGGG

### Plasma IgE and human lactoferrin specific‐IgE and IgG levels

The IgE levels in plasma were measured using a mouse IgE ELISA kit (Morinaga Institute of Biological Science, Yokohama, Japan). The levels were quantified by the standard in the kit and detection limit was 0.5 ng/mL.

In order to detect human lactoferrin specific‐IgE and IgG, we performed ELISA as described previously (Shibamori et al. 2006). Briefly, microwell plates were coated with 2 ng/100 *μ*L/well of human lactoferrin and incubated at 4°C overnight. After blocking with PBS containing 5% FBS and washing with PBS‐T, plasma samples diluted in PBS containing 5% FBS were added to microwell plates and incubated at 4°C overnight. Bound IgE and IgG were then reacted with HRP‐conjugated anti‐mouse IgE (eBioScience, San Diego, CA) or an IgG antibody (Abcam, Cambridge, MA) and TMP solution (Cosmobio, Tokyo, Japan). Colorimetric changes were measured by optical density (OD 450 nm). Results were expressed as relative units compared to the saline group.

### Immunochemical and histopathological evaluations

Immunostaining was performed as reported previously (Takemoto et al. 2007). Briefly, deparaffinized lung tissue sections were incubated for 30 min in methanol containing 1% H_2_O_2_ in order to inactivate endogenous peroxidase. After three washes in Tris‐buffered saline for 5 min, sections were incubated in 5% normal goat serum. Specimens were incubated at 4°C overnight with polyclonal antibodies against arginase I (1:50) and human lactoferrin (1:150). Specimens were then treated with goat anti‐rabbit immunoglobulin conjugated with a peroxidase‐labeled dextran polymer (Dako, North America, Carpinteria, CA) at room temperature for 1 h. Visualization was performed by adding 3,3‐diaminobenzidine tetrahydrochloride (Dako) as a substrate, and counterstaining was performed with hematoxylin. Rabbit non‐immunoglobulin (Dako) was used as a negative control.

In hematoxylin and eosin (H&E) staining, fixed lung tissues were dehydrated, embedded in paraffin, and sectioned to examine inflammation. Periodic acid‐Schiff (PAS) staining was also performed to assess goblet cell hyperplasia, and this was followed by Azan staining to visualize collagen fibers in interstitial spaces. The levels of inflammation in peribronchial areas of the lung were estimated as described in previous study (Murakami et al. 2015) using ImageJ software (National Institutes of Health, Bethesda, MD); A value of 0, no inflammation in bronchi; a value of 1, bronchi occasional infiltrated with inflammatory cells; a value of 2, most bronchi surrounded by a thin layer (1–5 cells thick) of inflammatory cells; and a value of 3, most bronchi surrounded by a thick layer (more than 5 cells thick) of inflammatory cells. Goblet cell hyperplasia and collagen fiber were semi‐quantitatively expressed using ratio of positive area by PAS or Azan staining in bronchial area.

### Purity of human lactoferrin by SDS‐PAGE and western blotting

The purity of human lactoferrin (50 ng) was assessed using SDS‐PAGE with detection by silver staining (Sigma) and transferal to PVDF membranes (Millipore, Billerica, MA). After blocking with 5% dried skimmed milk in Tris‐buffered saline containing 0.5% Tween 20 (TBS‐T), blots were incubated with rabbit anti‐human lactoferrin (1:1000) (Abcam) or mouse anti‐human lactoferrin IG in plasma of mice with human lactoferrin‐induced airway inflammation in this study (1:400) and a horseradish‐peroxidase (HRP)‐conjugated secondary antibody (1:2000 or 1:4000) (Dako). Antibody‐specific bands were detected using an enhanced chemiluminescence Western blot detection system (Perkin‐Elmer, Boston, MA).

### Statistical analysis

Statistical analyses of results with two types of independent variables were performed by a two‐way ANOVA (group × concentration) followed by Bonferroni's post hoc test. In order to compare differences in two sets of data with normal distributions, a two‐tailed unpaired *t*‐test was performed. If normality tests failed, the Mann–Whitney U‐test was used. Data are expressed as the mean ± standard error (SEM). *P* values < 0.05 were considered significant. GraphPad Prism (GraphPad Software, San Diego, CA) was used as software in statistical analyses.

## Results

### Effects of human lactoferrin on AHR

**Figure 2:**
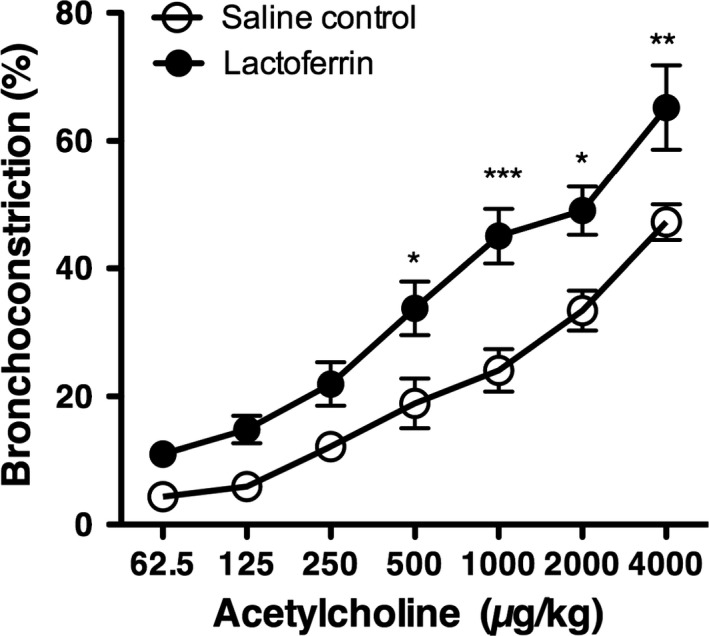
Airway hyperresponsiveness to acetylcholine after exposure to human lactoferrin. A two‐way ANOVA showed no interaction between the concentration of acetylcholine and treatment with lactoferrin. Bonferroni's post hoc test for multiple comparisons was performed on bronchoconstriction values in the saline control and lactoferrin groups for each concentration of acetylcholine (62.5, 125, 250, 500, 1000, and 2000 mg/kg). Values were expressed as the mean ± SEM of 4–6 mice. *P < 0.05, **P < 0.01, ***P < 0.001.

AHR values in the saline control group and lactoferrin group increased as ACh concentrations became higher. The two‐way ANOVA showed an interaction between the concentration of ACh and the treatment group. Therefore, a one‐way ANOVA was performed and revealed significant differences in bronchoconstriction values for AHR between the saline control group and lactoferrin group. Bonferroni's multiple comparison test as a post hoc analysis showed significantly higher bronchoconstriction values against 500 *μ*g/kg, 1000 *μ*g/kg, 2000 *μ*g/kg, and 4000 *μ*g/kg ACh in the lactoferrin group than in the saline group (Fig. 2).

### Effects of lactoferrin on BALF

**Figure 3:**
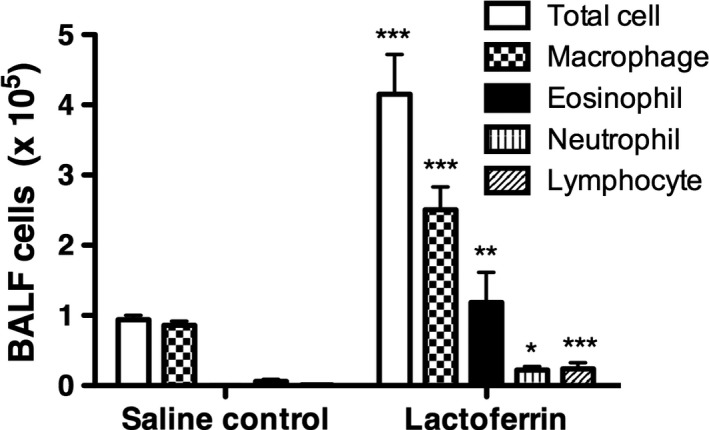
Bronchoalveolar fluid (BALF) cell numbers in total cell fractions, macrophages, eosinophils, neutrophils, and lymphocytes. BALF cell numbers were expressed as the mean ± SEM of seven mice. An unpaired *t*‐test was performed for each cell between the saline control and lactoferrin groups. *P < 0.05, **P < 0.01, and ***P < 0.001.

Total cell numbers and the percentages of macrophages, eosinophils, neutrophils, and lymphocytes in BALF were significantly higher in mouse groups inoculated with lactoferrin than in those exposed to saline (Fig. 3).

### Effects of lactoferrin on the mRNA expression of cytokines, chemokines, arginases, and nitric oxide synthases

**Figure 4:**
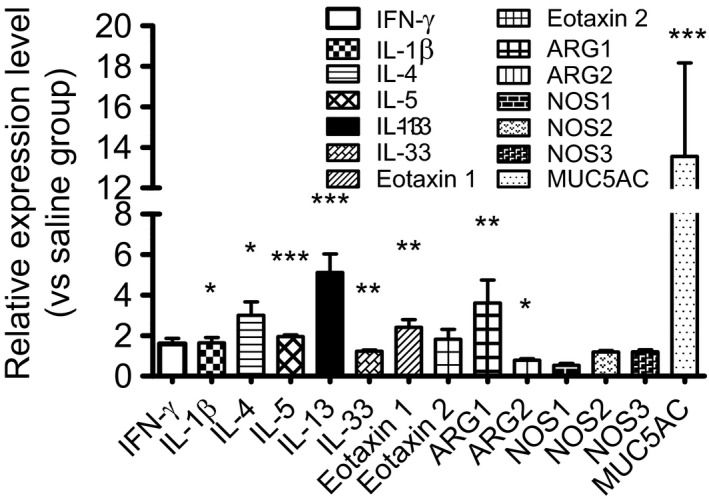
Changes in mRNA expression in lung tissue after exposure to human lactoferrin. Real‐time PCR for the cytokines IFN‐*γ*, IL‐1ß, IL‐4, IL‐5, IL‐13, and IL‐33, for the chemokines eotaxin 1 and eotaxin 2, and for arginase I (ARG1), arginase II (ARG2), nitric oxide synthase 1 (NOS1), nitric oxide synthase 2 (NOS2), and nitric oxide synthase 3 (NOS3) was performed under optimized conditions. Relative expression was calculated with GAPDH as an internal standard. Data are expressed as the mean ± SEM of seven mice. *P < 0.05, **P < 0.01, and ***P < 0.001 represent significant differences in an unpaired *t*‐test.

The mRNA expression levels of several cytokines, chemokines, arginase (I, II), nitric oxide synthase (1, 2, and 3), and MUC5AC were measured using real‐time PCR (Fig. 4). The mRNA expression levels of IL‐4, IL‐5, IL‐13, IL‐33, eotaxin 1, arginase I, arginase II, IL‐1*β*, and MUC5AC were significantly higher in the lactoferrin group than in the saline control group.

### Effects of lactoferrin on plasma IgE and lactoferrin‐specific IgG and IgE levels

**Figure 5:**
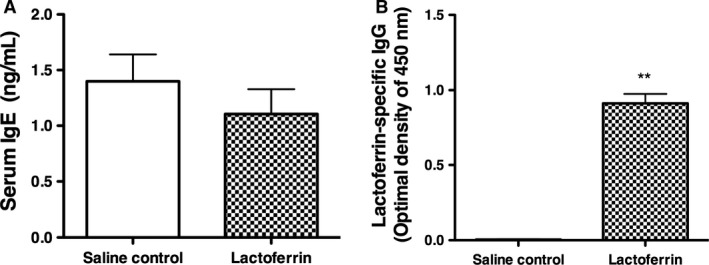
Plasma IgE and human lactoferrin‐specific IgG. No significant differences were observed in plasma IgE levels between the saline control and lactoferrin groups (A). Specific IgG against human lactoferrin was higher in mice with human lactoferrin‐induced airway inflammation than in control mice (B).

Total IgE levels were measured in the saline control group and lactoferrin group. No significant differences were observed in the plasma levels of IgE between the two groups (Fig. 5A). Specific IgE against lactoferrin was not detected in the lactoferrin or saline control group. However, specific IgG levels were significantly higher in the lactoferrin group than in the saline control group (Fig. 5B).

### Effects of lactoferrin on histopathological changes in the lung

**Figure 6:**
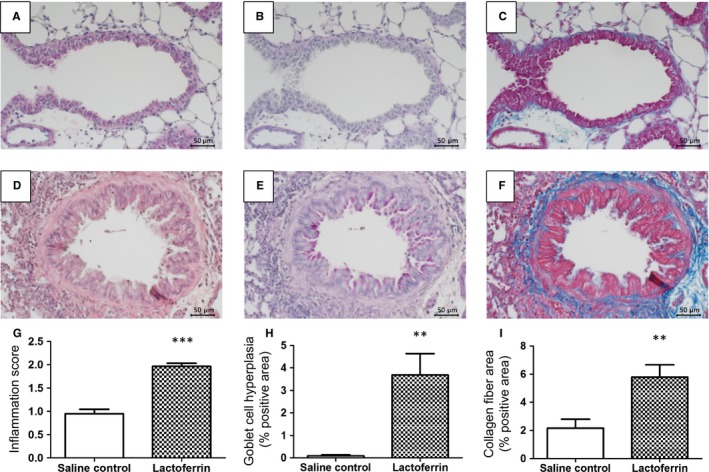
Histopathological findings of lungs exposed to human lactoferrin. Paraffin‐embedded sections were stained with hematoxylin‐eosin for the saline control (A) and lactoferrin (D) groups, with PAS for the saline control (B) and lactoferrin (E) groups, and with Azan for the saline (C) and lactoferrin (F) groups. Inflammation, goblet cell hyperplasia, and collagen fiber in bronchi were scored by ImageJ softwere (G–I). Data are expressed as the mean ± SEM of seven mice. **P < 0.01, and ***P < 0.001 represent significant differences in an unpaired *t*‐test.

Histopathological changes in lung tissue after the inoculation with lactoferrin were shown in Figure 6. The peribronchial accumulation of inflammatory cells in the lungs was more prominent in the lactoferrin group than in the saline control group (Fig. 6A, D and G). PAS staining for goblet cell hyperplasia in bronchial epithelial cells was more intense in the lactoferrin group than in the saline control group (Fig. 6B, E and H). The number of collagen fibers around the bronchus, as revealed by Azan staining, was higher in the lactoferrin group than in the saline control group (Fig. 6C, F and I).

**Figure 7:**
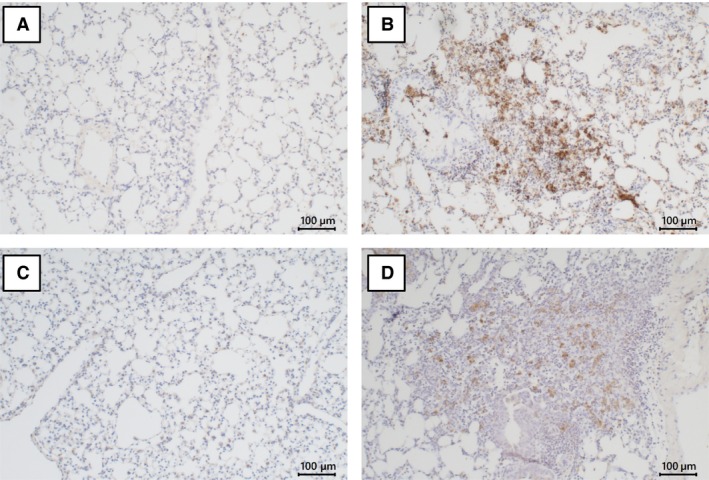
Immunohistochemical localization of human lactoferrin and arginase I. Immunostaining for human lactoferrin was observed in alveolar lining cells and macrophages. Immunostained cells were prominent in inflammatory granules in the lung (B). No staining for human lactoferrin was observed in the lungs of saline control mice (A). Immunostaining for arginase I was localized in inflammatory granules (D) and was weaker in the lungs of saline control mice (C).

The immunohistochemical localization of human lactoferrin was observed in accumulated macrophages in inflammatory sites, alveolar macrophages, and alveolar lining cells (Fig. 7B), while that of arginase I was observed in inflammatory macrophages (Fig. 7D).

### Purity of human lactoferrin by SDS‐PAGE and western blotting

**Figure 8:**
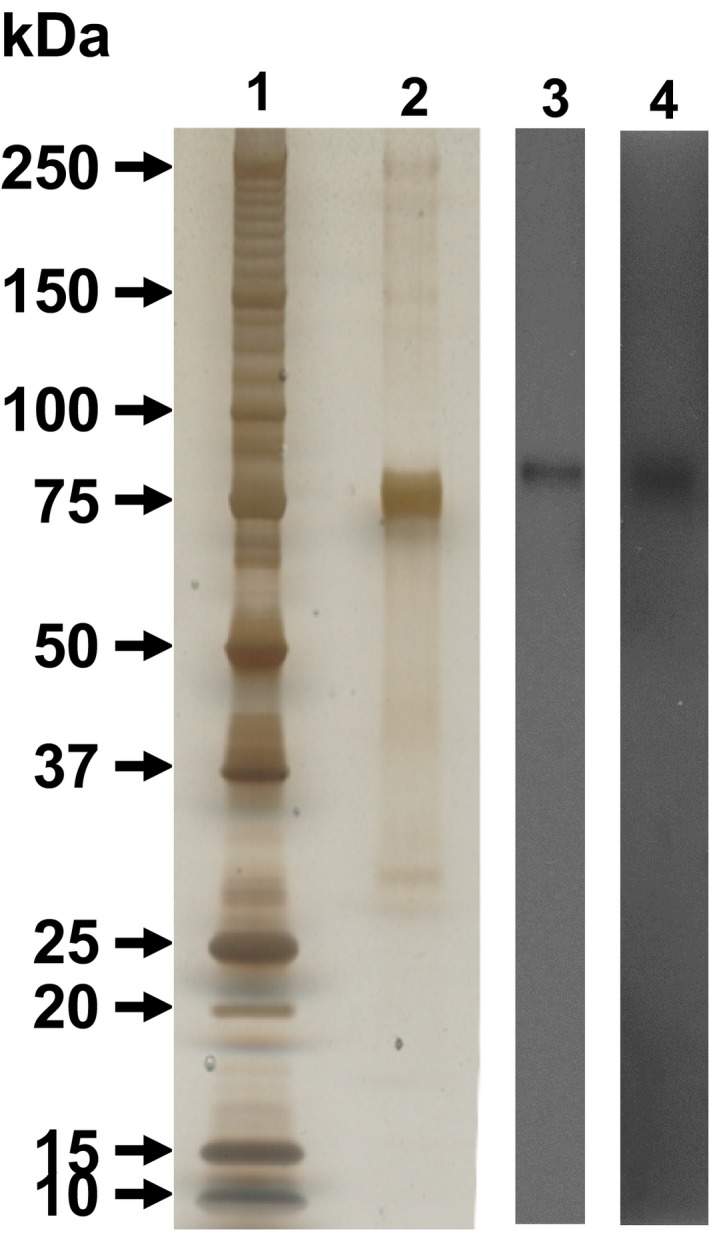
Purity of human lactoferrin and specificity of human lactoferrin‐specific IgG. The purity of human lactoferrin was assessed by SDS‐polyacrylamide gel electrophoresis (PAGE) and western blot. Lane 1 is molecular marker and lane 2 is silver stain of human lactoferrin (50 ng); lane 3 is SDS‐PAGE of human lactoferrin (50 ng) and following western blotting using anti‐human lactoferrin antibody; lane 4 is SDS‐PAGE of human lactoferrin (50 ng) and following western blotting using mouse plasma with human lactoferrin‐induced airway inflammation.

The identification of human lactoferrin was assessed by SDS‐PAGE and western blotting (Fig. 8). After the electrophoresis of 50 ng human lactoferrin, a single main protein band with a molecular weight of 80‐kDa was detected in lane 2. Purified lactoferrin could be confirmed by western blotting using rabbit polyclonal anti‐human lactoferrin and plasma from mice with human lactoferrin‐induced airway inflammation contained human lactoferrin‐specific IgG in lane 3 and 4, respectively.

## Discussion

The results of this study demonstrated for the first time that human lactoferrin induces allergic airway inflammation in NC/Nga mice. Lactoferrin is generally considered to ameliorate the severity of inflammation and allergies (Hill and Newburg 2015; Giansanti et al. 2016), and it has been reported that the single intranasal administration improved allergic rhinitis in mice (Wang et al. 2013). However, native human lactoferrin had antigenicity and generated specific IgG against human lactoferrin in mice, in which iron binding capacity or its glycosylation may contribute to its antigenicity (Almond et al. 2012, 2013) Lactoferrin from cow milk has been correlated with the severity of atopic dermatitis (Röckmann et al. 2014). Therefore, the induction of allergic airway inflammation by human lactoferrin was anticipated.

The oral administration of bovine lactoferrin was previously shown to induce its movement from the gastrointestinal lumen to various organs in mice such as the brain, liver, kidney, and spleen by overcoming major proteolytic degradation, and induced significantly high levels of bovine lactoferrin‐specific IgA and IgG (Fischer et al. 2007). Furthermore, the chronic feeding of bovine lactoferrin generated bovine‐lactoferrin‐containing IgA and IgG immune complexes. Immobilized lactoferrin, such as that in airway inflammatory cell surfaces, activates eosinophils, and this is followed by $O_{2}^{-}$ production, neurotoxin generation, and leukotriene C4 production via receptors for lactoferrin in eosinophils (Thomas et al. 2002). In this study, increases in the number of eosinophils in BALF may have been due to the up‐regulation of mRNA for eosinophil‐related chemokines and cytokines such as IL‐4, IL‐5, IL‐13, and eotaxin. Although the precise mechanisms responsible currently remain unclear, the continuous inoculation of lactoferrin into the airway lumen may activate eosinophils.

The immunohistochemical localization of human lactoferrin in the lungs of mice with human lactoferrin‐induced airway inflammation indicated that nasally administered human lactoferrin remains in alveolar lining cells and macrophages. There is a report that oral intake of bovine lactoferrin did not receive proteolytic process after intestinal absorption and generated immune complexes with lactoferrin‐specific IgG (Fischer et al. 2007). Therefore, it is not surprising that human lactoferrin was detected in alveolar lining cells and in alveolar macrophages in human lactoferrin‐induced airway inflammation in mice.

Arginase I was induced at the mRNA and protein levels in the lungs of mice with human lactoferrin‐induced airway inflammation. Previous studies reported that the strong induction of arginase I with *Dermatophagoides farinae* (DF)‐induced allergic airway inflammation may result in AHR (Takemoto et al. 2007; Takahashi et al. 2010). The induction of arginase I consumes L‐arginine, and, thus, the generation of nitric oxide from L‐arginine by nitric oxide synthase (NOS) for bronchial smooth muscle relaxation decreases because NOS uses L‐arginine as a common substrate with arginase. However, it currently remains unclear whether arginase is involved in the pathogenesis of human lactoferrin‐induced allergic airway inflammation.

Previous findings on DF‐induced airway inflammation implicated IL‐33, which regulates Th2 cytokines, in its pathogenesis (Murakami et al. 2015). IL‐33 mRNA was up‐regulated in this study; therefore, IL‐33 may be involved in the up‐regulation of IL‐13. The induction of arginase I is transcriptionally regulated by STAT6 that is under the control of IL‐13R/IL‐4R*α* signal transductions (Yang et al. 2006; Qin et al. 2016; Valladao et al. 2016). Therefore, although the precise mechanisms underlying the induction of IL‐13 and arginase I following an inoculation with lactoferrin remain unclear, human lactoferrin may stimulate IL‐13 and arginase I in the lungs of NC/Nga mice.

Although total IgE levels were not enhanced in this study, specific IgG levels for human lactoferrin increased. Moreover, mice with human lactoferrin‐induced airway inflammation produced immunoglobulin against human lactoferrin, as shown in Figure 8. Allergen‐specific IgG_1_ generally plays an important role in the activation and degranulation of eosinophils as well as in the manifestation of allergic disease (Kaneko et al. 1995; Oshiba et al. 1996).

In this study, we demonstrated for the first time that human lactoferrin induces allergic airway inflammation in NC/Nga mice. However, we attempted the procedure similar to a method using OVA, which is not a common allergen for human. These methods used alum adjuvant to induce immune responses. To indicate the possibility of allergic airway inflammation by lactoferrin, following experiments might be needed in the future: oral administration without alum adjuvant, mice strain differences in the pathogenesis of human lactoferrin‐induced airway inflammation, and comparative study using recombinant lactoferrin instead of human lactoferrin.

## Conflict of Interest

No potential conflict of interest relevant to this study was reported.
